# Validity of Two New Brief Instruments to Estimate Vegetable Intake in Adults

**DOI:** 10.3390/nu7085305

**Published:** 2015-08-11

**Authors:** Janine Wright, Jillian Sherriff, John Mamo, Jane Scott

**Affiliations:** School of Public Health, Curtin University, Perth 6102, Australia; E-Mails: jane.scott@curtin.edu.au (J.S.); j.mamo@curtin.edu.au (J.M.); j.sherriff@curtin.edu.au (J.S.)

**Keywords:** vegetable, brief instrument, validity, dietary assessment, dietary monitoring

## Abstract

Cost effective population-based monitoring tools are needed for nutritional surveillance and interventions. The aim was to evaluate the relative validity of two new brief instruments (three item: VEG3 and five item: VEG5) for estimating usual total vegetable intake in comparison to a 7-day dietary record (7DDR). Sixty-four Australian adult volunteers aged 30 to 69 years (30 males, mean age ± SD 56.3 ± 9.2 years and 34 female mean age ± SD 55.3 ± 10.0 years). Pearson correlations between 7DDR and VEG3 and VEG5 were modest, at 0.50 and 0.56, respectively. VEG3 significantly (*p* < 0.001) underestimated mean vegetable intake compared to 7DDR measures (2.9 ± 1.3 *vs.* 3.6 ± 1.6 serves/day, respectively), whereas mean vegetable intake assessed by VEG5 did not differ from 7DDR measures (3.3 ± 1.5 *vs.* 3.6 ± 1.6 serves/day). VEG5 was also able to correctly identify 95%, 88% and 75% of those subjects not consuming five, four and three serves/day of vegetables according to their 7DDR classification. VEG5, but not VEG3, can estimate usual total vegetable intake of population groups and had superior performance to VEG3 in identifying those not meeting different levels of vegetable intake. VEG5, a brief instrument, shows measurement characteristics useful for population-based monitoring and intervention targeting.

## 1. Introduction

A diet high in fruit and vegetable intake is known to decrease risk of chronic diseases including coronary heart disease CHD [1,2,3] and some cancers [4]. The World Health Organization recommends people eat at least 400 grams of fruit and vegetables daily (excluding potatoes) [1]. Increasing the intake of fruit and vegetables is a world-wide public health priority [5] with most countries having culturally-specific dietary recommendations to increase fruit and vegetables, which require monitoring [6,7]. There is a need for cost effective population-based monitoring tools for the purposes of nutritional surveillance and the targeting of nutrition interventions. Traditional dietary intake assessment methods such as 24 h recalls (24HR), diet records and long-form food frequency questionnaires (FFQ) are not suitable for many population monitoring and intervention settings as they are resource intensive, costly, and involve high respondent burden. Some short dietary questions [6] and abbreviated dietary intake assessment instruments have been developed [6,8] and are being increasingly used for both population-based monitoring [6] and assessing the impact of interventions [9].

Abbreviated instruments, used to estimate vegetable intake and assess other dietary behaviours, do not replace more detailed and comprehensive methods for measuring overall dietary intake such as diet records and 24HR but can provide valuable information on food group specific dietary behavior and level of consumption for population groups [6,10]. Brief instruments have advantages in situations where due to resource or time constraints it is not feasible to use more detailed measures of dietary intake [11]. However, the measurement characteristics of brief instruments should be considered and they should be used in appropriate contexts. Brief instruments with questions on self-reported fruit and vegetable consumption have been shown to be able to assess group mean/median intakes [6,8,10], a characteristic useful for monitoring and for identifying intervention target populations [10].

Brief dietary intake assessment instruments have a specific and limited focus [6]. This means that these instruments can be designed to specifically measure the population dietary behaviours of interest and relevance, an aspect particularly useful for targeting of behavioral-based interventions. For instance, a simple 1-item summary question on usual total vegetable intake in cups or serves has been routinely used in national and regional Australian studies [6,12] and has been widely used in the United States [12]. However, when evaluated this question was found to be valid only in discriminating between groups with significantly different intakes [6]. Furthermore, when used within a 2-item fruit and vegetable screener, this vegetable consumption question was found to underestimate median values for vegetables when compared to multiple 24HR [12]. Thus, in the area of vegetable intake estimation, there is a need to develop and assess the validity of alternative vegetable consumption questions or brief question sets [6,10,12].

The design of alternative questions for assessing vegetable consumption used in this study is informed by research evidence which indicates where improvements to questions could lead to useful and meaningful instruments with greater validity. The concept of what a serving size of vegetables is has been shown to be a factor adding to question difficulty in brief instruments [13] with Yarcoh *et al.* [12] recently reporting better performance of a 2-item fruit and vegetable screener that asked about intake in cups compared to intake in servings. Other difficulties in vegetable consumption questions relate to vegetable use in mixed dishes (e.g., vegetable soups) [8,10], the presence of multiple vegetable preparation forms (e.g., salads, cooked vegetables) [14], and the difficulty in interpreting questions which ask for the exclusion and inclusion of different types of potatoes [13]. Furthermore, it has been determined that it is desirable that vegetable consumption questions have less restrictive response categories [6], and in line with current dietary guidance [15] would also be able to assess consumption of vegetable sub-groups such as starchy vegetables and red and orange colored vegetables.

VEG3 and VEG5 are two new brief instruments for estimating usual total vegetable intake, consisting of three and five items respectively. The instruments were generated from six different questions on vegetable consumption, with five of these vegetable consumption questions having design aspects which address some of the identified challenges in estimating vegetable intake. This may lead to better measurement characteristics of these brief instruments. The aim of this paper is to assess the relative validity of VEG3 and VEG5 to estimate usual total vegetable intake for population monitoring and intervention targeting purposes through comparison to vegetable intake measured by a 7-day estimated dietary record (7DDR).

## 2. Methods

### 2.1. Participants

Sixty-four volunteers aged between 30 and 69 years were recruited. Recruitment was by newspaper and community announcements including a general practitioner (GP) surgery advertisement. Inclusion criteria were men and women aged between 30 and 69 years requiring primary or secondary prevention of cardio-vascular disease (CVD), that is, having one or more CVD risk factors. Exclusion criteria were: persons with non-insulin dependent diabetes; non-English speaking; unable to read or write; currently undertaking major dietary modification; major intercurrent illness. Eligibility was assessed by self-report via telephone interview. All participants provided written informed consent and the study was approved by the Curtin University of Technology Human Ethics Committee, approval reference number HR20/98.

### 2.2. Study Design

At baseline, subjects completed (1) a socio-demographic questionnaire; (2) a 63-item FFQ [16,17,18] containing the six vegetable consumption questions from which the 3-item (VEG3) and 5-item (VEG5) instruments are derived, and (3) were given instruction to commence the 7-day estimated diet record the following day. Four weeks later, subjects completed (1) a questionnaire on any dietary changes since baseline measures and (2) the second administration of the 63-item FFQ containing the vegetable consumption questions that make up VEG3 and VEG5. All materials were mailed to participants. Relative validity was determined through comparison of total vegetable intake estimates calculated from the first administration of VEG3 and VEG5 to total vegetable intake measured from the 7-day estimated dietary records. Test-retest reliability was determined through comparison of total vegetable intake estimates between the two administrations of VEG3 and VEG5 (at baseline and four weeks later) in those not reporting dietary changes in the intervening period.

### 2.3. Study Tools

#### 2.3.1. Seven-Day Estimated Dietary Records (7DDR)

The reference dietary assessment method was a 7-day estimated dietary record (7DDR) including food-photo serving size description aids, modified with permission from those used and validated by Raats and Geekie [19]. The booklet consisted of two pages of instructions, a sample day record, 12 blank pages for recording food and drinks and six pages of photographs depicting reference “medium” portion sizes of foods . The booklet also included two fold-out flaps with descriptions of “medium” portion sizes. Participants were asked to describe the “amount” eaten in terms of photographs and lists provided, in terms of household measures and weights taken from food packaging. Participants received telephone-based training on how to complete the 7DDR with phone call assistance available during the 7-day measuring period.

#### 2.3.2. Brief Instrument (VEG3 and VEG5) Vegetable Consumption Questions

**Figure 1:**
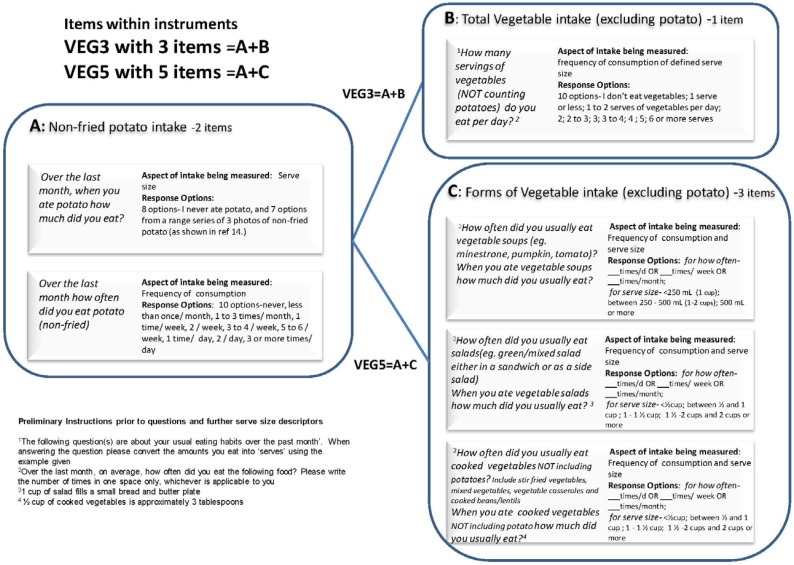
Vegetable consumption question details for VEG3 and VEG5.

VEG3 and VEG5 were derived from six questions assessing vegetable consumption contained within a longer, validated 63-item combination FFQ [16,17,18]. The six questions were Ling and colleague’s [14] 3-item set of questions (identified as C on Figure 1) on the consumption of vegetable soups, salads and cooked vegetables (excluding potato); a 1-item summary question (B on Figure 1) on usual total vegetable consumption (excluding potato); and two questions on non-fried potato intake (A on Figure 1) (the format and calculation of non-fried potato using these questions is further described in [18]). The time period of reference for all vegetable consumption questions was the previous month. Details of the questions and how they are combined to create the brief instruments are shown in Figure 1 with VEG3 = A + B and VEG5 = A + C.

### 2.4. Data Analysis

#### 2.4.1. Dietary Analysis

Food groups were defined according to the specifications of the Australian Guide to Healthy Eating [20]. Food group data were determined through export of FoodWorks diet record food lists (FoodWorks Version 3.0 (Xyris software, Brisbane , QLD, utlilising the NUTTAB98 database ) into Microsoft Access (Microsoft Access 2007, Microsoft Enterprise Systems 2007) where all individual food and drink items were coded into food groups. This food group coding was then re-linked to each participant’s dietary record to allow calculation of 7-day food group totals (total serves) and daily averages (servings/day). Food group outcome variables for vegetables were average daily serves of vegetables with and without non fried potato.

#### 2.4.2. Brief Instrument (VEG3 and VEG5) Vegetable Intake Estimation

Total usual vegetable intake estimates were calculated using two different sets of questions as previously described and outlined in Figure 1. Both brief instruments were also used to calculate usual total vegetable intake excluding potato. For this the two questions related to non-fried potato intake were removed, in which case VEG3 had 1-item (B on Figure 1) and VEG5 had 3-items (the question set developed by Ling *et al.* [14] and shown as C on Figure 1).

### 2.5. Statistical Analysis

The relationship between the 7DDR measures with VEG3 and VEG5 estimates was assessed using Pearson’s correlation coefficients. The ability of the brief instruments VEG3 and VEG5 to estimate group means for usual total vegetable intake (both including and excluding non-fried potatoes) in comparison to 7DDR measures was investigated using paired t-tests. The technique of Bland and Altman [21] was utlilised to determine agreement between the 7DDR and brief instrument vegetable intake estimates, and to calculate mean bias and 95% limits of agreement (LOA). To examine how well the brief instruments could correctly classify subjects according to different intake levels the 7DDR, VEG3 and VEG5 measures were dichotomized for different serve intake levels (for example less than five servings/day or five or more servings/day), with data dichotomized four times in total (allowing examination of classification of intake from two to five serves/day). The predictive value of the VEG3 and VEG5 (both including and excluding non-fried potato) was examined using these dichotomized scores, with positive predictive value defined as those determined to be consuming less than the intake level serving/day cut-off by the brief instruments who also did not meet the same intake level serving/day cut-off according to the 7DDR measure. Test-retest reliability of the short instruments was assessed using Pearson’s correlation coefficient. All data were analyzed using the Statistical Package for the Social Sciences (SPSS Version 17.0, Chicago, IL, USA). All data distributions (7DDR, VEG3 and VEG5) were checked for normality using a Shapiro-Wilk test. *p* < 0.05 was considered significant.

## 3. Results

### 3.1. Subject Characteristics

The mean age of the 64 adult volunteers was mean age ± SD 55.7 ± 9.6 years (30 males, mean age ± SD 56.3 ± 9.2 years and 34 female mean age ± SD 55.3 ± 10.0 years).

### 3.2. Test-Retest Reliability

Out of the 56 who completed the vegetable consumption questions making up VEG3 and VEG5 on two occasions four to six weeks apart , 19 reported making dietary changes in that period and thus a total of 37 persons (20 male mean ± SD age, 56.6 ± 9.1 years, 17 female mean age 56.5 ± 9.4) were included in an assessment of test-retest reliability. Total vegetable intake at baseline and at one month re-administration for VEG3 were mean ± SD 3.1 ± 1.4 and 3.1 ± 1.0 serves/day; and for VEG5 were 3.7 ± 1.3 and 3.1 ± 1.3 serves/day. The test-retest correlation coefficients between one-month re-administrations of VEG3 and VEG5 for total vegetable intake in serves/day were, for VEG3, *r* = 0.64 95% CI (0.40,0.80) and for VEG5, *r* = 0.58 95% CI (0.31,0.76), with both indicating reasonable to good test-retest reliability.

### 3.3. Relative Validity of Brief Instruments in Comparison to Dietary Record Measures

Pearson correlation coefficients between 7DDR measure of vegetable intake in serves/day and VEG3 and VEG5 estimates were modest at *r* = 0.50 95% CI (0.29, 0.66) and *r* = 0.56 95% CI (0.36, 0.71), respectively. For VEG3 the mean difference (7DDR-VEG3) or bias was 0.63 serves/day with 95% LOA of (−2.20, 3.45 serves/day). For VEG5 the mean difference (7DDR-VEG5) or bias was 0.24 serves/day with 95% LOA of (−2.57, 3.04 serves/day).

As shown in Table 1, VEG3 significantly (*p* < 0.001) underestimated mean vegetable intake compared to 7DDR measures (2.9 ± 1.3 *vs.* 3.6 ± 1.6 serves/day, respectively), whereas mean vegetable intake assessed by VEG5 did not differ from 7DDR measures (3.3 ± 1.5 *vs.* 3.6 ± 1.6 serves/day, respectively).

These differences between VEG3 and VEG5 were mirrored in prevalence estimates for intakes meeting different national food selection guide-specified serve intake levels (Table 1). VEG5 produced prevalence estimates of individuals meeting different intake levels (*i.e.*, from less than two serves to more than five serves) that were similar to 7DDR measures, with VEG3 produced prevalence estimates being somewhat different. VEG5 was able to correctly identify 91% of those not meeting the recommendation of five or more serves per day according to the 7DDR, and could correctly identify 71% and 72% of those not consuming at least four or three serves a day, respectively. When non-fried potato was excluded from VEG5, it identified 95%, 88% and 75% of those not consuming five, four, and three serves a day according to their 7DDR classification. VEG5 had superior positive predictive values to VEG3, the exception being for the less than five serves/day comparison point (95% *vs.* 97%).

## 4. Discussion

Wide 95% LOA for both VEG3 and VEG5 estimates of usual total vegetable intake in comparison to 7DDR measures indicates poor agreement between the brief instruments and the reference standard at the individual level. As such, neither VEG3 nor VEG5 can replace the comprehensive dietary measure of 7DDR in estimating vegetable intakes of individuals.

However, when assessing the brief instruments’ estimation of vegetable intake at the group level, VEG5’s estimation of mean vegetable intake did not differ from that measured by the 7DDR, whereas the VEG3’s vegetable intake estimates at group level were significantly underestimated. Both of these results were found irrespective of whether non-fried potato was included or excluded in the estimation of vegetable intake. This result is consistent with the finding of the Kim and Holowaty review [10] in that the longer instrument had better relative validity than the shorter one.

These results indicate that VEG5, as an estimate of vegetable intake both including and excluding non-fried potato, is an instrument that could be used to assess mean vegetable intake in population groups. Although this study is smaller and has less generalizability than that of Yaroch and colleagues [12], their 16-item screener (with 12 vegetable consumption questions) was unable to estimate group mean vegetable intake.

VEG5 had superior positive predictive values to VEG3, the exception being for the less than five serves/day comparison point (95% *vs.* 97%). These high positive predictive values suggest that VEG5, in particular, is useful for confirming intake levels and will correctly classify sizeable and similar proportions of adults with vegetable intakes below, at or above three, four and five servings per day.

**Table 1:** Estimation of usual total vegetable intake by VEG3 and VEG5 in comparison to 7-day estimated dietary records (7DDR) measures of mean vegetable intake, and predictive value of VEG3 and VEG5 for different vegetable intake levels.

		<5 serves/day		<4 serves/day		<3 serves/day		<2 serves/day	
		Prevalence ^b^, %	Positive Predictive Value	Prevalence, %	Positive Predictive Value	Prevalence, %	Positive Predictive Value	Prevalence, %	Positive Predictive Value
*Vegetable measures including potato (non-fried)*									
All (*n* = 64)	Vegetable serves/day ^a^								
7DDR	3.6 ± 1.6 (3.2,4.0)	87.5	…	65.6	…	37.5	…	12.5	…
VEG3	2.9 ± 1.3 * (2.6,3.3)	92.2	0.90	81.2	0.68	53.1	0.59	26.6	0.29
VEG5	3.3 ± 1.5 (3.0,3.7)	87.5	0.91	60.9	0.71	39.1	0.72	17.2	0.45
*Vegetable measures excluding potato*									
All (*n* = 64)	Vegetable serves/day ^a^								
7DDR	3.0 ± 1.5 (2.7,3.4)	93.8	…	82.8	…	51.6	…	21.9	…
VEG3-potato	2.5 ± 1.1 * (2.2,2.7)	93.8	0.97	92.2	0.85	64.1	0.63	31.3	0.35
VEG5-potato	2.8 ± 1.3 (2.5,3.2)	96.9	0.95	79.7	0.88	50.0	0.75	23.2	0.40

^a^ mean ± SD (95% CI); * *p* ≤ 0.05 in comparison to 7DDR; ^b^ population prevalence estimates below each vegetable intake level using 7DDR, VEG3 or VEG5.

Overall, VEG5 shows measurement characteristics useful for population-level monitoring and for the targeting of interventions. VEG5 can identify those with intakes less than dietary recommendations, and was also able to identify and classify most people with consumption levels considerably lower than current recommendations. This maximizes potential uses of VEG5 in intervention targeting, as most consumers have intakes less than three serves a day [7,22] and represent the group that can make the greatest public health gains through increased consumption towards dietary recommendations [7].

Furthermore, VEG5 has demonstrated characteristics useful for population-level monitoring independent of whether non-fried potato is included or excluded in the calculation of usual total vegetable intake. Thus VEG5 can be used in nations which differ in their treatment of non-fried potatoes in their vegetable intake recommendations; for example, Australia includes non-fried potatoes within their definition of total vegetable intake [7] whereas the US does not [15].

As a point of difference to many currently used short dietary questions and brief instruments, VEG5 includes open-ended response options for frequency estimates of consumption of soups, salad and cooked (non-potato) vegetables. VEG5 can therefore collect information on the full range of intakes including high vegetable consumers. VEG5 estimates may therefore remain relevant to monitoring contexts when and if the recommendations for vegetable intake increase from those currently used.

In this study, relative validity, rather than absolute validity, was assessed with only self-reported data compared. The use of the 7DDR as the reference standard is, however, a robust comparison method: the 7DDR is a comprehensive dietary intake measure which, importantly, has measurement errors most likely independent from the main systematic measurement errors common to brief instruments, which are the cognitive task of estimating usual intake over a period of time [11]. Also, the relative validity testing in this study did not test the vegetable consumption questions that are part of VEG3 and VEG5 as stand-alone items; rather the question items were administered as part of a longer 63-item FFQ. The impact of this is not known, but as potential applications of VEG5 include population monitoring, it is likely that in these settings, the brief instruments questions will also be used in combination with other dietary and health questions. The findings of this study were demonstrated with a relatively small volunteer sample size and further studies with larger and more diverse population groupings are needed to examine the generalisability of the findings.

## 5. Conclusions

When compared to the results from 7DDR, a 5-item set of questions (VEG5) better assessed vegetable intake of adult Australians at the group-level than a three item set of questions (VEG3)*.* Both VEG3 and VEG5 were able to correctly classify high proportions of those subjects not consuming five, four and three serves/day of vegetables according to their 7DDR classification. Neither VEG3 nor VEG5 were accurate in assessing vegetable intake at an individual level and do not replace more thorough and comprehensive dietary intake measurement tools such as diet records for this purpose. VEG5 is a quick, inexpensive instrument that can assess mean intake of vegetables in population groups and can identify those not meeting public health-related vegetable consumption recommendations. Specifically, VEG5 has high predictive value in identifying those not consuming three serves to five serves of vegetables a day. These useful characteristics of VEG5 apply whether non-fried potato is included or excluded from its vegetable intake estimates. As a 5-item instrument to estimate usual total vegetable intake, VEG5 therefore appears useful for population-level monitoring and intervention targeting purposes.

## References

[1] Organization W.H. (2003). Report of WHO/FAO Expert Consultation. Diet, Nutrition and the Prevention of Chronic Diseases.

[2] Dauchet L., Amouyel P., Hercberg S., Dallongeville J. (2006). Fruit and vegetable consumption and risk of coronary heart disease: A meta-analysis of cohort studies. J. Nutr..

[3] Pomerleau J., Lock K., McKee M. (2006). The burden of cardiovascular disease and cancer attributable to low fruit and vegetable intake in the European union: Differences between old and new member states. Public Health Nutr..

[4] World Cancer Research Fund/American Institute of Cancer Research (2007). Food, Nutrition, Physical Activity, and the Prevention of Cancer: A Global Perspective.

[5] World Health Organization (2003). World Health Assembly Resolution Wha57.17-Global Strategy on Diet, Physical Activity and Health.

[6] Coles-Rutishauser I., Webb K., Abraham B.L., Allsopp R. (2001). Evaluation of short dietary questions from the 1995 National Nutrition Survey.

[7] Pollard C.M., Miller M.R., Daly A.M., Crouchley K.E., O’Donoghue K.J., Lang A.J., Binns C.W. (2008). Increasing fruit and vegetable consumption: Success of the Western Australian go for 2&5 campaign. Public Health Nutr..

[8] Thompson F., Subar A., Smith A., Midthune D., Radimer K., Kahle L., Kipnis V. (2002). Fruit and vegetable assessment: Performance of 2 new short instruments and a food frequency questionnaire. J. Am. Diet. Assoc..

[9] Pollard C., Miller M., Woodman R.J., Meng R., Binns C. (2009). Changes in knowledge, beliefs, and behaviors related to fruit and vegetable consumption among Western Australian adults from 1995 to 2004. Am. J. Public Health.

[10] Kim D.J., Holowaty E.J. (2003). Brief, validated survey instruments for the measurement of fruit and vegetable intakes in adults: A review. Prev. Med..

[11] Kirkpatrick S.I., Reedy J., Butler E.N., Dodd K.W., Subar A.F., Thompson F.E., McKinnon R.A. (2014). Dietary assessment in food environment research: A systematic review. Am. J. Prev. Med..

[12] Yaroch A.L., Tooze J., Thompson F.E., Blanck H.M., Thompson O.M., Colón-Ramos U., Shaikh A.R., McNutt S., Nebeling L.C. (2012). Evaluation of three short dietary instruments to assess fruit and vegetable intake: The national cancer institute’s food attitudes and behaviors survey. J. Acad. Nutr. Diet..

[13] Wolfe W.S., Frongillo E.A., Cassano P.A. (2001). Evaluating brief measures of fruit and vegetable consumption frequency and variety: Cognition, interpretation, and other measurement issues. J. Am. Diet. Assoc..

[14] Ling A.M., Horwath C., Parnell W. (1998). Validation of a short food frequency questionnaire to assess consumption of cereal foods, fruit and vegetables in Chinese Singaporeans. Eur. J. Clin. Nutr..

[15] U.S. Department of Agriculture and U.S. Department of Health and Human Services (2010). Dietary Guidelines for Americans.

[16] Barkess J.L., Sherriff J.L. (2003). Relative Validity and Reliability of a Short Food Frequency Questionnaire to Assess Saturated Fat Intake Behaviours.

[17] Barkess J.L., Sherriff J.L. Relative validity of an Australian short food frequency questionnaire to assess intake of fruit, vegetables and cereal foods. Proceedings of the XIV International Congress of Dietetics.

[18] Wright J., Sherriff J., Dhaliwal S., Mamo J. (2011). Tailored, iterative, printed dietary feedback is as effective as group education in improving dietary behaviours: Results from a randomised control trial in middle-aged adults with cardiovascular risk factors. Int. J. Behav. Nutr. Phys. Act..

[19] Raats M.M., Sparks P., Geekie M.A., Shepherd R. (1999). The effects of providing personalized dietary feedback. A semi-computerized approach. Patient Educ. Counsel..

[20] Smith A., Kellett E., Schmerlaib Y. (1998). Australian Guide to Healthy Eating.

[21] Martin Bland J., Altman D.G. (1986). Statistical methods for assessing agreement between two methods of clinical measurement. Lancet.

[22] Australian Bureau of Statistics 4364.0.55.003—Australian Health Survey: Updated Results, 2011–2012. http://www.abs.gov.au/ausstats/abs@.nsf/Lookup/C549D4433F6B74D7CA257B8200179569?opendocument.

